# Magnetic Nanoparticles Embedded in a Silicon Matrix

**DOI:** 10.3390/ma4050908

**Published:** 2011-05-17

**Authors:** Petra Granitzer, Klemens Rumpf

**Affiliations:** Institute of Physics, Karl Franzens University Graz, Universitaetsplatz 5, A-8010 Graz, Austria; E-Mail: klemens.rumpf@uni-graz.at

**Keywords:** porous silicon, magnetic nanoparticles, nanocomposite

## Abstract

This paper represents a short overview of nanocomposites consisting of magnetic nanoparticles incorporated into the pores of a porous silicon matrix by two different methods. On the one hand, nickel is electrochemically deposited whereas the nanoparticles are precipitated on the pore walls. The size of these particles is between 2 and 6 nm. These particles cover the pore walls and form a tube-like arrangement. On the other hand, rather well monodispersed iron oxide nanoparticles, of 5 and 8 nm respectively, are infiltrated into the pores. From their size the particles would be superparamagnetic if isolated but due to magnetic interactions between them, ordering of magnetic moments occurs below a blocking temperature and thus the composite system displays a ferromagnetic behavior. This transition temperature of the nanocomposite can be varied by changing the filling factor of the particles within the pores. Thus samples with magnetic properties which are variable in a broad range can be achieved, which renders this composite system interesting not only for basic research but also for applications, especially because of the silicon base material which makes it possible for today’s process technology.

## 1. Introduction

Magnetic nanoparticles are used in various research fields such as e.g., physics, chemistry, material science and biomedicine, in which the main interest is to obtain knowledge about the novel physical and chemical properties which appear if an object comes into the nanometer scale. Surface effects and proximity effects become more important and so-called quantum size effects are responsible for the unusual behavior of such nanosized materials compared to their bulk materials. The fabrication of isolated magnetic nanoparticles is not easy due to the dramatic influence of oxidation, because of the large surface areas compared to their volume and because of the tendency of metallic nanoparticles to agglomerate. To overcome this problem, particles are capped by an outer shell of a few nanometers which is often a metal-oxide [1], silica [2] or a surfactant [3]. The presence of such a shell excludes magnetic exchange interactions and thus only dipolar coupling contributes to the magnetic behavior. A further method to stabilize nanoparticles is the use of a matrix, in which polymers [4], SiO_2_ [5] or zeolites [6] are often employed as substrate. Concerning the magnetic behavior, the employment of a matrix gives an additional degree of freedom and allows tuning the properties depending on the particle arrangement within the matrix.

Magnetic nanoparticles are of interest for high density data storage but also for biomedical applications. Due to interactions between nanosized magnetic systems, new properties arise such as soft magnetic alloys [7] and hard magnetic materials with an improved energy product [8].

Homo-(Co, Fe, Ni) and heterometallic (FeCo, FeCu, Co–Cu) magnetic nanoparticles can be prepared from a metal salt by using a reducing agent such as complex hydrides [9]. Pure metal particles are not stable in air and, due to oxidation, they change or lose their magnetic behavior. The magnetic properties depend on the degree of oxidation, whereas a complete suppression of oxidation during the fabrication process cannot be guaranteed. As a further protection to avoid agglomeration of the particles, an organic shell is formed around the particles. To achieve an oxide layer (e.g., Co–O) covering the metal core (e.g., Co) which exhibits antiferromagnetic behavior, the organic protective coating is removed [9] and by exposing the particles to air an oxide layer is formed. In such ferromagnetic/antiferromagnetic layered systems, exchange bias effects [10] are investigated. Another method of particle stabilization is the incorporation of the particles within a non-magnetic matrix which prohibits agglomeration and oxidation and ensures the properties of the individual particles.

Further prominent and versatile applicable particles, are iron oxide particles whereas magnetite is of interest not only because of the magnetic behavior but also because of the low toxicity and thus the applicability in biomedicine. One common method is the fabrication by thermal decomposition at high temperatures of an iron organic precursor using an organic medium [11]. This method enables the fabrication of nanoparticles in a wide size range depending on the precursors, solvents and surfactants. In using a low precursor concentration and low boiling temperature and a Fe/oleic acid ratio of 1:1, small particles between 4 and 6 nm can be produced [12]. The size can be varied by changing the surfactant or the Fe/surfactant ratio, wherein the particle size increases with an increase of this ratio [12]. The shape of the particles can be modified by changing the precursor, its concentration or by adding impurities during the fabrication process [12]. If such particles are synthesized with an organic shell, agglomeration is prohibited.

One crucial point of nanoparticle utilization is its application in biomedicine. In this case their encapsulation to render them biocompatible is of importance, but also adequate coating of the particles for attaching them to antibodies, proteins or drugs is of interest. A further approach is the coating of nanoparticles with anti-tumorigenic substances to target the tumor site without affecting the healthy tissue [13]. To overcome the disadvantage of affecting the body and to treat the center of disease more specifically, distinct antibodies are conjugated to the nanoparticles to selectively bind them to related receptors and inhibit tumor growth [14]. The transport of magnetic particles within the human body to a specific target and the localization of the particles need the application of an external magnetic field. In the case of *in vivo* applications, low magnetic fields are desirable and thus particles offering a high magnetization at room temperature are under consideration. Also the encapsulation of magnetic nanoparticles within a matrix for controlled drug delivery, is of interest. Porous silicon which offers bio-behavior [15] and low toxicity is an attractive material for such an application. The combination of porous silicon with magnetic particles, namely Fe_3_O_4_, and additional loading with a molecular payload is of interest for controlled transport in applying an external magnetic field. The loaded molecules (enzymes) can be transported and subsequently released in an appropriate solution [16]. The fabrication of a porous silicon double-layer of different pore-size is used for loading with magnetite nanoparticles and a small amount of liquid. The samples can be heated within an oscillating magnetic field, due to the superparamagnetic magnetite nanoparticles (~10 nm in size) [17]. The application of porous silicon as matrix is not only suitable because of its biocompatibility but also because its optical properties can be used for identification. For example microporous silicon offers light emitting properties in a visible range and macroporous silicon etched as photonic crystal provides a unique spectral code [17].

The incorporation of nanoparticles into matrices is of interest not only because of the stabilization of the particles but also because of the resulting magnetic properties which can be influenced by the filling factor of the particles but also by the morphology of the matrices.

Porous silicon, which can be fabricated by anodization of a silicon wafer in hydrofluoric acid solution in various morphologies [18], can be used as matrix for the incorporation of nanoparticles. It offers pores in different size regimes, depending on the doping of the wafer as well as on the anodization conditions. Magnetite particles are in solution and thus are incorporated by infiltration into the pores. Another possible procedure is electrodeposition of a metal salt solution into the pores and the precipitation of metal nanoparticles. These two different methods of particle incorporation within the pores of porous silicon will be discussed in the following.

## 2. Nanocomposite System

### 2.1. Fabrication of the Porous Silicon Matrix

**Figure 1:**
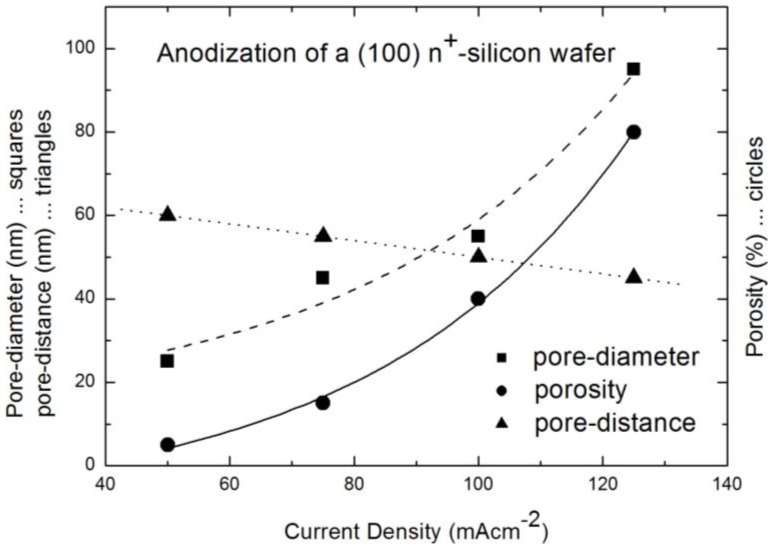
Relation between current density and pore-diameter (squares), distance between the pores (triangles) and porosity (circles) (data from [19]).

Porous silicon matrices which are used for the incorporation of magnetic nanoparticles are fabricated by anodisation of a highly n-doped (10^19^ cm^−3^) silicon wafer. As electrolyte aqueous hydrdofluoric acid (10 wt% HF) is used. The aim is to get oriented pores, grown perpendicular to the surface and with a quite narrow pore-diameter distribution. In using the same doping density of the wafer, the pore-diameter can be modified by varying the anodization conditions. An increase of the applied current density results in an increase of the pore-diameter and a concomitant decrease of the distance between the pores (Figure 1).

By choosing the etching parameters in an adequate way (current densities around 100 mAcm^−2^), the arrangement of the pores is quasi-regular and the size distribution of the pore-diameter is quite narrow which means the deviation at full width at half maximum (FWHM) is less than 10% [20]. In the case of the employment of porous silicon as template for magnetic nanoparticles, straight and separated pores are of importance. In using a (100) silicon wafer the main growth direction of the pores is perpendicular to the surface, whereas the pores also offer a dendritic growth with small side-pores in (111) direction. The growth of the side-pores is favored by the remaining free carriers in the remaining silicon skeleton. The length of the side pores is less than half the distance between the pores and thus a clear separation of the pores is guaranteed. For higher current densities, the critical current density J_PS_ is reached and instead of pore formation, electropolishing at the porous silicon/silicon border takes place. By decreasing the current density the pore arrangement and the size distribution becomes more irregular and the length of the side pores increases. In the case of smaller pore-diameters (<35 nm) the distance between the pores becomes greater than twice the thickness of the space charge region, which allows the nucleation of pores in these remaining silicon walls. By coming below a certain diameter (<10 nm) the pores are not oriented any more, but offer a network-like structure.

### 2.2. Fabrication of the Iron Oxide Nanoparticles

There are various methods to produce iron oxide nanoparticles of different sizes, whereas a well established procedure to fabricate magnetite nanoparticles is thermal decomposition at high temperatures [11]. Magnetite nanoparticles of 5 and 8 nm have been fabricated at the Instiute of Material Science in Madrid, CSIC, in using the method of high temperature decomposition. Magnetite nanoparticles have been synthesized using iron acetylacetonate as precursor and phenyl ether as solvent. A mixture of 0.71 g of Fe(acac)_3_ (2 mmol), 2.38 g of 1,2-hexadecanediol (10 mmol), 1.69 g of oleic acid (6 mmol), 1.60 g of oleylamine (6 mmol) and 20 ml of trioctylamine have been added to a three-neck flask. Then, the mixture has been heated under mechanical stirring and a flow of nitrogen gas until a temperature of 200 °C has been reached. This temperature has been kept constant for 120 min and then the solution has been heated to reflux (369 °C) for 30 min in a nitrogen atmosphere. Finally, the solution has been cooled down to room temperature.

**Figure 2:**
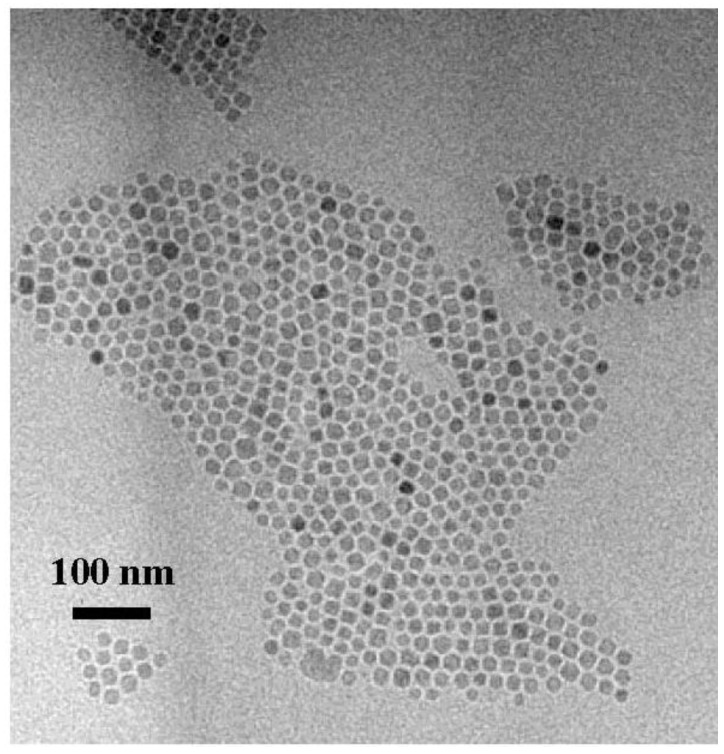
TEM-image of magnetite particles of 8 nm in size and an oleic acid coating of about 2 nm.

The powder was obtained by precipitation with ethanol, collected with a magnet and finally dried under nitrogen flow. A stable suspension of the powder could be obtained when nanoparticles have been mixed with 20 mL of hexane and 0.05 mL of oleic acid and sonicated for a time period of 5 min. The achieved particles show quite a monodispersed size-distribution of 8 nm obtained from TEM-images (Figure 2) with a deviation of ±1.5 nm. To gain the size-distribution of the particles images taken from the distribution of the Fe-content have been used to improve the contrast of the TEM-images [21].

### 2.3. Incorporation of Magnetic Nanoparticles into the Pores of Porous Silicon

Magnetic nanoparticles can be produced, for example by various physical or chemical routes [22], to obtain particles with a specific size and shape. One method is electrodeposition from a metal salt solution. In this case, homo- and heterometallic particles can be obtained as a powder. To fill the pores of porous silicon matrices with magnetic nanoparticles a ferromagnetic metal such as Ni is electrodeposited within the pores resulting in a composite material consisting of a silicon matrix and precipitated Ni nanostructures. The deposition is performed in a pulsed way under cathodic conditions. The pulsed current is applied to suppress the exhaustion of the electrolyte along the pore-length and to guarantee that the metal ions can reach the pore-bottom. In general, electrodeposition is influenced by charge transfer as well as by mass transport of the reactants, whereas mass transport, the dominant component, occurs at the micrometer scale and therefore is difficult to control in the case of nanoscale structures [23]. The metal deposition within the pores of porous silicon with diameters around 60 nm is performed in choosing the parameters in such a way that the limiting pulse current density is not reached and thus a capacity effect can be neglected. This condition is reached if the pulse duration is less than the charging/discharging time of the electrochemical double layer. If the pulse current is high, the thickness of the double layer becomes small [24]. As electrolyte an adequate metal salt solution is employed. In the case of Ni-deposition the so called Watts electrolyte (NiCl_2_, NiSO_4_) with boric acid as buffer is used. Typically a solution of 45 g/L NiCl_2_, 300 g/L NiSO_4_ and 45 g/L H_3_BO_3_ is used. A variation of the pulse frequencies and the current density leads to modifications of the geometry and spatial distribution of the metal deposits within the pores [25]. A decrease of the pulse duration from from 40 s to 5 s and keeping the current density constant at 25 mA/cm^2^, leads to an increase of the elongation of the Ni-structures (spherical particles of about 50 nm in diameter up to wire-like structures of a few micrometers in length) [26] which is summarized in Table 1. A variation of the current density influences mainly the spatial distribution of the deposits [27].

**Figure 3:**
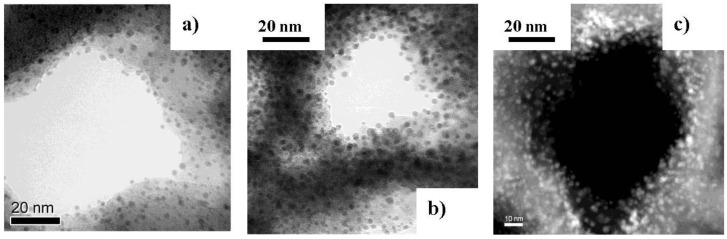
Ni-nanoparticles between 2 and 6 nm electrodeposited on the pore walls of porous silicon matrices. An increase of the deposition current density from (**a**) 25; (**b**) 35 to (**c**) 50 mAcm^−2^ results in closer packing of the particles, forming a metal-tube like arrangement [34].

Ni particles between 2 and 6 nm in size are precipitated along the pore walls. An increase of the current density from 25 mA/cm^2^ to 50 mA/cm^2^ leads to a decrease of the distance between the particles. The pulse duration has been kept constant at 20 s (Figure 3).

**Table 1:** In this table the electrochemical deposition parameters such as applied current density and pulse duration of the current for various geometries of the Ni-nanostructures are summarized.

	Current density [mA/cm^2^]	Pulse duration [s]
**Wires** (up to a few µm)	25	5
**Ellipsoids** (up to 500 nm)	25	10
**Sphere-like particles** (~50 nm)	25	40
**Small particles** (2–6 nm)	25 to 50	20

The work of H. Sato *et al*. [28] deals with the deposition of Ni within the pores of porous silicon in using a single electrolyte composed of hydrofluoric acid (HF) for silicon dissolution as well as a Ni-salt solution as Ni^2+^ source for the Ni deposition. Due to the fact that n-type silicon was used as substrate material, the back side of the samples had to be illuminated during the process to generate holes for the silicon etching. Other groups report on the electrodeposition of various metals into porous silicon, such as Cu, Pt Pd, Au [29], but also the deposition of magnetic materials, such as Co and Fe and their alloys [30], has been studied.

A further approach to incorporate magnetic materials within a matrix is metallo-organic chemical vapor deposition (MOCVD) [31,32] or atomic layer epitaxy (ALE) [33].

Magnetite nanoparticle solutions containing particles of 5 and 8 nm in size are infiltrated into the pores of porous silicon matrices. First the porous silicon has been dried to remove the electrolyte completely from the pores. The anodization electrolyte contains ethanol which would attack the oleic acid shell of the nanoparticles. During this drying process in ambient air, the hydrogen terminated porous silicon surface oxidizes and thus the pores are covered by a thin native oxide layer. The Fe_3_O_4_-anoparticle infiltration is carried out at room temperature for about 30 minutes.

The morphology of the achieved nanocomposite has been investigated by scanning and transmission electron microscopy (SEM, TEM). To see the spatial distribution of the particles within the pores, on the one hand SEM-images of cross-sectional regions are used and on the other hand the samples are investigated by TEM to get more details. But in the latter case, modifications of the sample by the preparation by focused ion beam (FIB) occur. EELS-spectra are used to obtain accurate information about the chemical composition of the system. For knowledge of the magnetic behavior, SQUID magnetometry (Cryogenics) and a vibrating sample magnetometer (PPMS, Quantum Design) are used.

## 3. Results and Discussion

**Figure 4:**
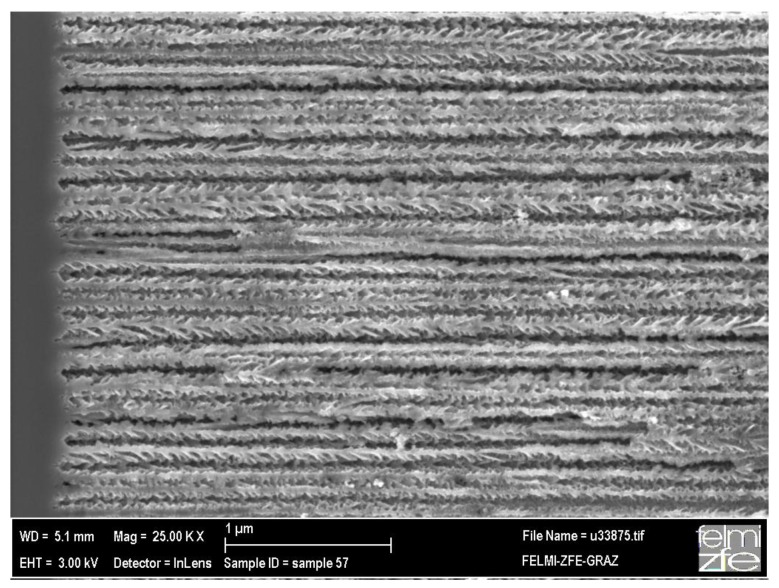
Cross-sectional scanning electron micrograph showing the pores with a diameter of about 60 nm. On the left hand side of the image the transition between porous silicon and bulk silicon can be seen.

The structure of the nanocomposite system and also the nature of the interface between the silicon skeleton and the incorporated magnetic nanoparticles is characterized by electron microscopy, whereas the morphology of the porous silicon templates is investigated by SEM. Figure 4 shows a cross-sectional SEM image of a typical porous silicon matrix with average pore-diameters of 60 nm and a mean distance of 40 nm between them.

**Figure 5:**
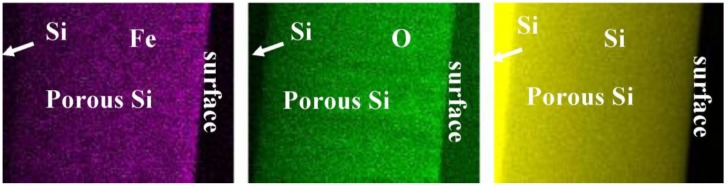
EDX mapping of the cross-sectional region of a porous silicon with infiltrated iron oxide nanoparticles. One sees that the distribution of the particles is quite homogeneous over the entire porous layer. From left to right: distribution of Fe (violet), oxygen (green) and silicon (yellow).

In using the back scattered electrons (BSE) elemental sensitive information is gained [35]. A survey of the spatial distribution of the magnetic particles within the porous silicon layer can be identified by energy dispersive X-ray (EDX) mapping (Figure 5).

**Figure 6:**
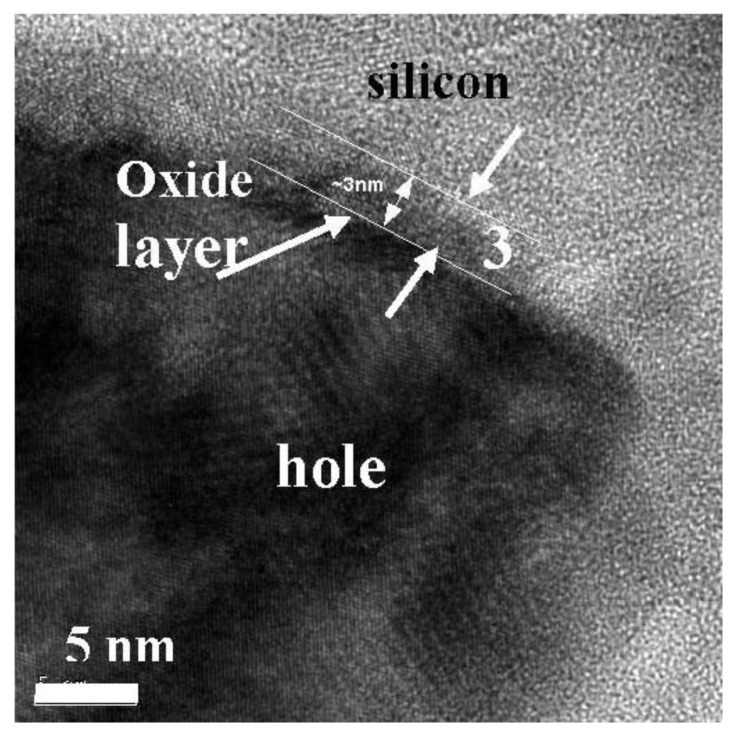
HR-TEM image of a porous silicon matrix which has been aged in air for 3 days. The pore walls are covered by a native oxide layer of about 3 nm.

Also, the interface between the remaining silicon matrix and the incorporated nanoparticles has been investigated by TEM. After the anodization of the silicon, the surface of the porous structure is hydrogen terminated. If the samples are aged in ambient air, a thin native oxide layer of about 3 nm is obtained which can be seen in Figure 6. Electron energy loss (EELS) spectra taken at the remaining silicon skeleton, show a high silicon peak around 200 eV and a low oxygen peak around 520 eV (not shown here).

If the electrodeposited Ni particles are oxidized during the deposition process, it is not yet clear whether the oxidation is due to the aging of the samples or due to the preparation by FIB.

### 3.1. Magnetic Characterization of Porous Silicon with Infiltrated Fe_3_O_4_ Nanoparticles

**Figure 7:**
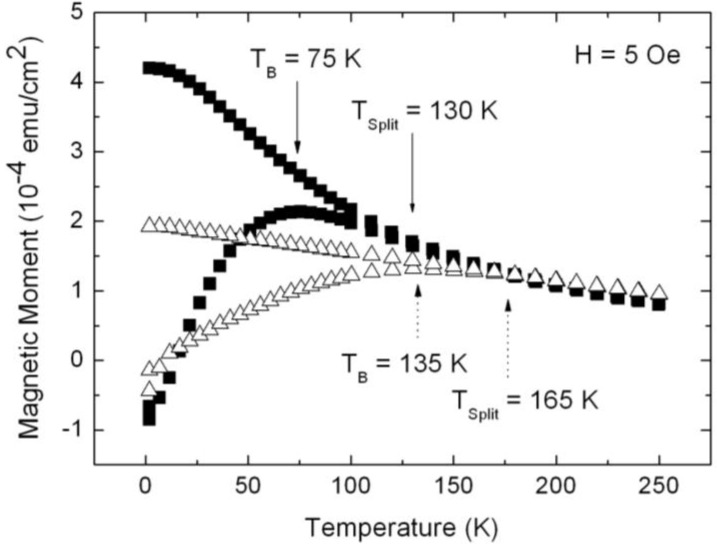
Temperature dependent magnetization measurements performed on samples with equal porous silicon morphology (pore-diameter ~90 nm, distance between the pores ~35 nm) infiltrated with two different concentrations of the iron oxide particle solution (triangles, initial concentration; squares, half of initial concentration). The average particle-size is 8 nm [37].

The magnetic nanocomposite is achieved, with porous silicon acting as substrate and infiltrated magnetite nanoparticles within the pores, leading to a system which shows a ferromagnetic behavior at low temperatures (T < T_B_) and superparamagnetism at higher temperatures (T > T_B_). This transition temperature can be influenced not only by the particle size [36] but also by the distance between the particles, which can be modified by changing the concentration of the particle-solution (Figure 7). Furthermore, the slope of the zero field cooled (ZFC)/field cooled (FC) curves changed. The more pronounced peak of the ZFC-branch, as well as the steeper slope of the curves of samples infiltrated with a solution of lower concentration, indicate a smaller dipolar interaction than in samples prepared with a magnetite solution of higher concentration.

**Figure 8:**
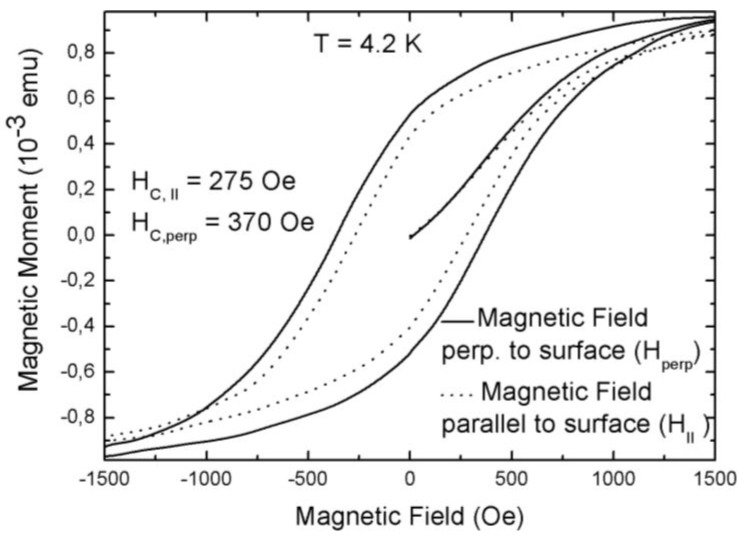
Field dependent magnetization measurements performed with a magnetic field applied parallel and perpendicular to the pores, respectively (pore-diameter ~60 nm, pore-distance ~40 nm, particle-size ~8 nm). A magnetic anisotropy (H_C, ⊥_ = 370 Oe, H_C, II_ = 275 Oe) between the two coercivities is observed due to dipolar coupling between the particles which is stronger within the pores than between particles of adjacent pores.

This distance between the nanoparticles can be varied by the coating used which influences the magnetic particle–particle interaction within the individual pores. Furthermore, the interaction between particles of adjacent pores can be modified by the thickness of the remaining silicon matrix, which influences the anisotropic behavior between magnetization measurements with an external field applied parallel and perpendicular to the sample surface (Figure 8).

**Figure 9:**
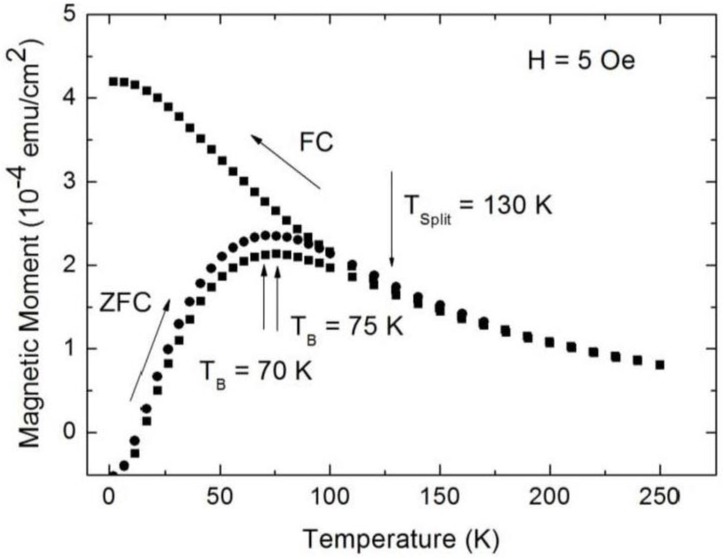
Zero field cooled/field cooled measurements performed in two directions, namely with a magnetic field applied parallel to the pores (circles) and perpendicular to the pores (squares). A small variation of T_B_ can be seen. (Particle size 8 nm; pore-diameter ~50 nm, pore-distance ~50 nm).

This anisotropy is caused by dipolar coupling of the magnetite particles which arises differently in the two magnetization directions. The minimum distance between the particles within the pores is twice the thickness of the coating, which is about 4 nm, whereas the minimum distance between particles of adjacent pores is the thickness of the pore-walls, which averages 40 nm. Thus the coupling between the particles within the pores is stronger than between particles of neighboring pores which displays anisotropic behavior. Temperature dependent magnetization curves measured in these two directions show a small shift of T_B_ (Figure 9). In the case of larger pore-diameters and smaller pore-distances this behavior becomes weaker, which results in a diminished anisotropy of the magnetization between the two directions.

**Figure 10:**
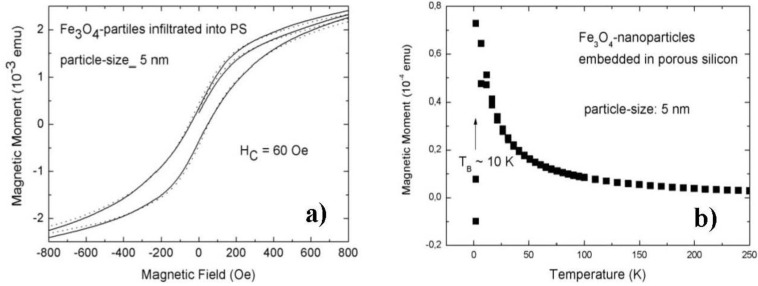
(**a**) Magnetization of porous silicon (pore-diameter ~50 nm, pore-distance ~50 nm) with embedded magnetite nanoparticles of 5 nm in size. This sample exhibits no magnetic anisotropy between the two magnetization directions perpendicular (full line) and parallel (dotted line) to the surface; (**b**) Zero field/field cooled magnetization of porous silicon with the same morphology with infiltrated 5 nm magnetite nanoparticles shows a blocking temperature (T_B_) of about 10 K (Figure 10 b taken from [38]).

Infiltration of magnetite nanoparticles of 5 nm in size into a matrix with pore-diameters of about 50 nm and a mean distance between the pores of 50 nm shows an isotropic behaviour between both directions of magnetization (Figure 10a). Furthermore the blocking temperature (T_B_) which indicates the change of regime between a superparamagnetic and a blocked state is around 10 K (Figure 10b) which is significantly decreased compared to the one of specimens with infiltrated particles of 8 nm. In the latter case, T_B_ is around 75 K for the same morphology of the matrix.

Both, the isotropic behavior as well as the low blocking temperature indicates that the 5 nm particles are not interacting. The dipolar coupling of nanoparticles is strongly correlated to the size of the particles and decreases linearly with the size. The oleic acid coating is for both particle sizes around 2 nm, whereas in the case of the 5 nm particles this separation has a stronger influence due to a weaker dipolar coupling of the smaller particles.

**Figure 11:**
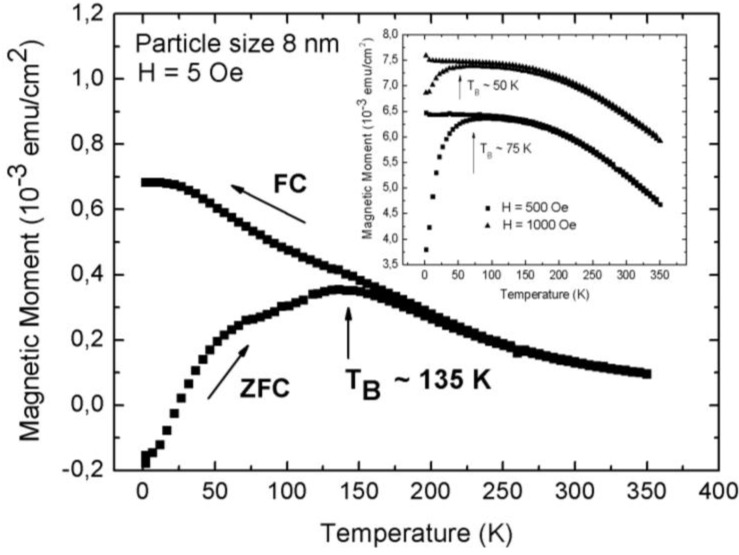
Zero field cooled (ZFC)/field cooled (FC) measurements of Fe_3_O_4_ particles infiltrated into porous silicon, carried out at a small applied magnetic field of 5 Oe and in a temperature range of between 4 K and 360 K. The blocking temperature (maximum peak of the ZFC-branch) at 135 K indicates dipolar coupling between the particles. Inset: Shift of T_B_ towards lower temperatures with increasing applied magnetic field from about 135 K (H = 5 Oe) to 75 K (H = 500 Oe) and 50 K (H = 1,000 Oe) [43].

The superparamagnetic behavior of the magnetite/porous silicon system above a blocking temperature T_B_ is shown by temperature dependent magnetization measurements. For specimens with an average pore-diameter of 90 nm, a mean distance between the pores of 35 nm and magnetite particles of 8 nm, zero field cooled (ZFC)/field cooled (FC) investigations performed at an applied field of 5 Oe, show a rather high blocking temperature T_B_ at 135 K, which indicates magnetic interactions between the particles (Figure 11). Similar findings of the blocking behavior of nanoparticles are reported in [39]. Furthermore, a shift of the blocking temperature to lower temperatures with higher applied fields is observed (inset Figure 11). This behavior of superparamagnetic particles is known to be proportional to H^2/3^, with H being the magnetic field, at high fields and proportional to H^2^ for lower fields [40].

Considering the ZFC/FC measurements, one recognizes that the splitting temperature between the ZFC and the FC-branch differs from the blocking temperature, which coincides with the maximum of the ZFC-curve. Such a behavior is observed in randomly dipolar coupled nanomagnet-systems [41]. Also, the width of the peak of the ZFC-branch can be attributed to dipolar coupling of the nanoparticles, since the distribution of the particle-size is quite well monodispersed, as proven by TEM-images (Figure 2) and analysis of the particle size distribution (not shown here).

The transition between superparamagnetic and blocked state occurs when the thermal energy barrier *KV* becomes equal to *25* *k_B_T* [42] where *K* is the anisotropy energy per unit volume of the superparamagnetic particle and *V* is its volume. For particles exhibiting a monodisperse size distribution the blocking temperature *T_B_* gives the threshold between the stable (*T < T_B_*) and unstable region (*T > T_B_*).

The particle size is known from TEM-images (average 8 nm) and thus the blocking temperature *T_B_* can be estimated by the equation *KV = 25* *k_B_T_B_*. By taking *K* = 1.35 × 10^4^ Jm^−3^ for Fe_3_O_4_ [44], this equation yields a value of *T_B_* = 10 K by assuming a spherical shape of the particles. This estimated value of *T_B_* is far away from the measured blocking temperature of 135 K by ZFC/FC measurements (Figure 11). An implication is the loss of validity of the used equation for non-interacting particles, thus magnetic interactions (dipolar) between the iron oxide particles can be concluded. Consequently it can be said that the particles of the investigated samples behave superparamagnetic only above a quite high blocking temperature of 135 K due to the presence of dipolar interactions. Kechrakos and Trohidou [45,46,47] and Allia *et al*. [48] showed that the blocking temperature is always enhanced due to interactions between the magnetic particles because the coupling suppresses the thermal fluctuations of the spins. Also a broadening of the ZFC peak is observed due to magnetic interactions [48]. The higher measured blocking temperature of about 135 K than the one estimated from the equation above (*T_B_* = 10 K) which is no longer valid for the ensemble of magnetically interacting particles, was investigated.

**Figure 12:**
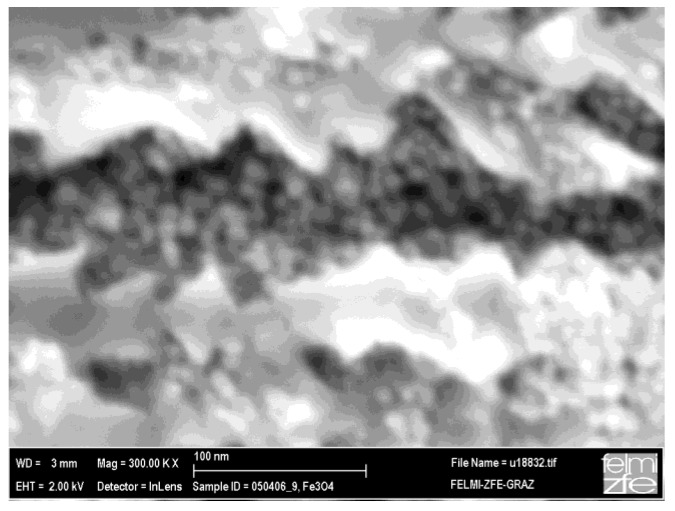
SEM image showing the individual iron oxide nanoparticles of about 8 nm in size within the pores of the porous silicon matrix [38].

Considering the magnetization curve of magnetite nanoparticles (size 8 nm) without PS-matrix, one sees that the blocking temperature is 160 K and the bifurcation of the ZFC and FC branches takes place at 230 K (not shown here). These temperatures are higher than in the case of magnetite nanoparticles embedded in porous silicon (PS). Given that in both cases the same particles are used, a reduction in the magnetic interaction can be assumed to take place when the magnetite nanoparticles are incorporated within the PS-matrix, which is plausibly caused by the morphology of the PS, exhibiting a distance between the pores (thickness of the remaining silicon pore-walls) of a few ten nanometers. Thus the interaction among the Fe_3_O_4_-nanoparticles, in one direction, along the pores is dominant. Moreover, due to the presence of oleic acid coating of the particles, exchange interaction is discarded and the interaction is mainly dipolar. Figure 12 shows a scanning electron micrograph of the cross section of a magnetite filled porous silicon sample. The nanoparticles within the pores have an average diameter of 8 nm. The distance between the particles within one pore is given by the coating being a few nanometers, whereas the distance between adjacent pores is given by the morphology of the porous silicon matrix which is in this case about 40 nm (more than 10 times greater than the mean particle-particle distance given by the surfactant). A more accurate investigation of the particles inside the pores by TEM failed due to the preparation by FIB and the concomitant sputtering and loosening of the particles.

Considering the hystereses loops of a porous silicon specimen infiltrated with magnetite nanoparticles, the magnetic anisotropy between the two magnetization directions offers coercivities of H_C_ = 370 Oe for an applied field parallel to the pores and H_C_ = 275 Oe for the field applied perpendicular to the pores (Figure 8). This anisotropic behavior differs drastically from the magnetization curve of a bare silicon wafer with the surface covered with the same iron oxide nanoparticles (not shown here) and using the same concentration of the magnetite-solution. In this latter case, the observed magnetic behavior is similar to the one of a thin magnetic film. The coercivities obtained for the two different magnetization directions for magnetite infiltrated into the pores vary which also shows that the particles are mainly incorporated within the pores and they do not only accumulate on the surface of the PS-template. The interaction between the particles occurs mainly within one pore (minimum distance between the particles is twice the thickness of the coating, 4 nm) but less from one pore to another one (average distance between adjacent pores is in the range of a few ten nanometers, depending on the sample preparation). The porous silicon specimens offering straight pores grown perpendicular to the surface are used as matrix for the Fe_3_O_4_ nanoparticles and influence the magnetic behavior to a great extent. Also the blocking temperature of the porous silicon/magnetite nanocomposite indicates the transition between superparamagnetic behavior and a blocked state is shifted to lower temperatures, compared to the equal bare Fe_3_O_4_ particles.

Magnetite is nowadays investigated because of its promising applications in nanomedicine for the location and diagnosis of tumors. Due to the biodegradability and biocompatibility of porous silicon [49] a combination of these two materials is a promising candidate for medical *in vivo* applications. The magnetic properties of the nanoparticle/PS system are of interest due to the magnetic phase transition controlled by the strength of the magnetic interaction, which is determined by the distance between the particles and the direction given by the matrix. Strongly interacting particles could lead to a blocking even at room temperature.

### 3.2. Magnetic Characterization of Porous Silicon with Deposited Ni Nanoparticles

Many works deal with the deposition (electrochemical or electroless) of ferromagnetic metals into non-magnetic templates, such as porous alumina [50,51,52] and porous silicon [53,54] to fabricate arrays of elongated nanostructures and nanowires. The formation of Ni nanotubes has been reported by Ogata *et al*. [55], whereas in this work Ni has been precipitated on the pore walls of p-type silicon under illumination which is necessary for the generation of electrons during the deposition process on the pore walls. Other works deal with the fabrication of metal nanotubes within porous alumina templates by atomic layer deposition (ALD) [56] or special sol-gel methods [57].

The metal/semiconductor composite which is achieved by a two electrochemical step process, wherein both the pore formation of the porous silicon matrices as well as the metal precipitation are self-organized, offers a ferromagnetic behavior, even though the precipitated metal particles are only a few nanometer in size (2–6 nm). The morphology of the porous silicon matrices can be varied in pore-diameter, pore-distance and pore-length as already mentioned in the previous chapters. Also in this case of electrodeposition of metal nanoparticles, a clear separation of the pores is of importance. In the case of precipitation of small nanoparticles on the pore walls, the spatial distribution can be modified by varying the current density above 25 mA/cm^2^. An increase of the current density from 25 to 50 mAcm^−2^ leads to more densely packed particle arrangements. Figure 3 shows the variation of the packing density of the particles covering the pore walls with respect to the applied current density.

**Figure 13:**
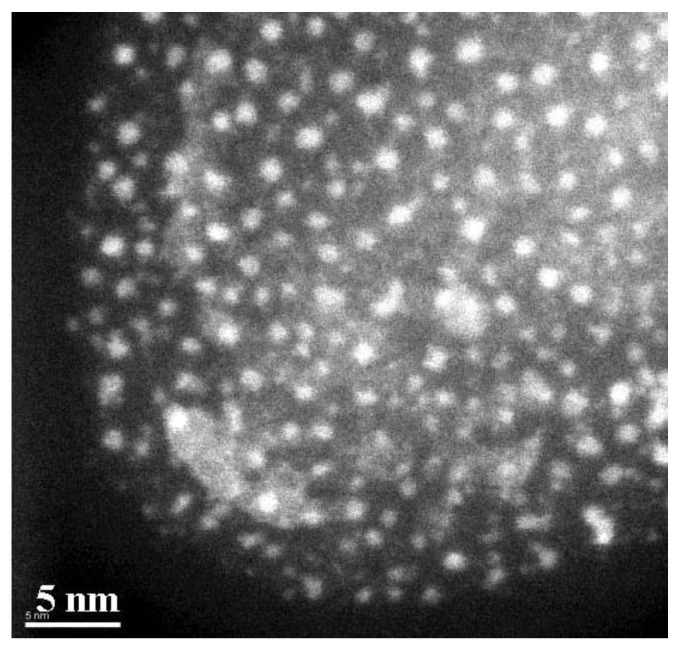
HRTEM image of a cross-sectional view of a pore showing the small Ni-particles precipitated at the pore-wall [34].

**Figure 14:**
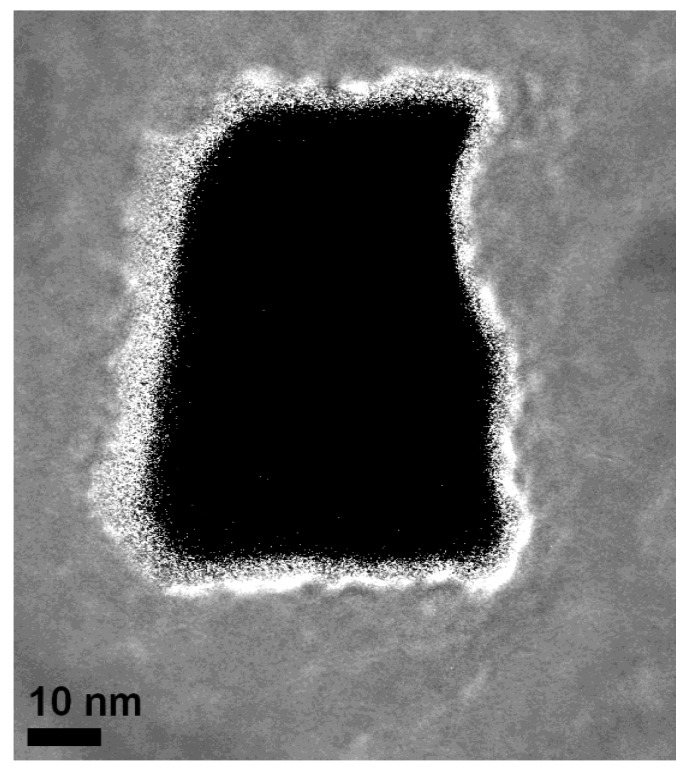
The tube-like arrangement of the closely packed Ni-particles can be seen in this plan-view transmission electron micrograph of an individual pore. The thickness of the Ni-tube is about 5 nm [34].

An accurate analysis of this tube-like arrangement at the pore-tips by high resolution transmission electron microscopy (HRTEM) (Figure 13) shows that the distribution of the Ni-particles is quite narrow, which means that the distance between the particles is smaller than 10 nm. In Figure 14 a plan-view image of an individual pore shows a Ni-nanotube with a wall-thickness of about 5 nm covering the pore.

**Figure 15:**
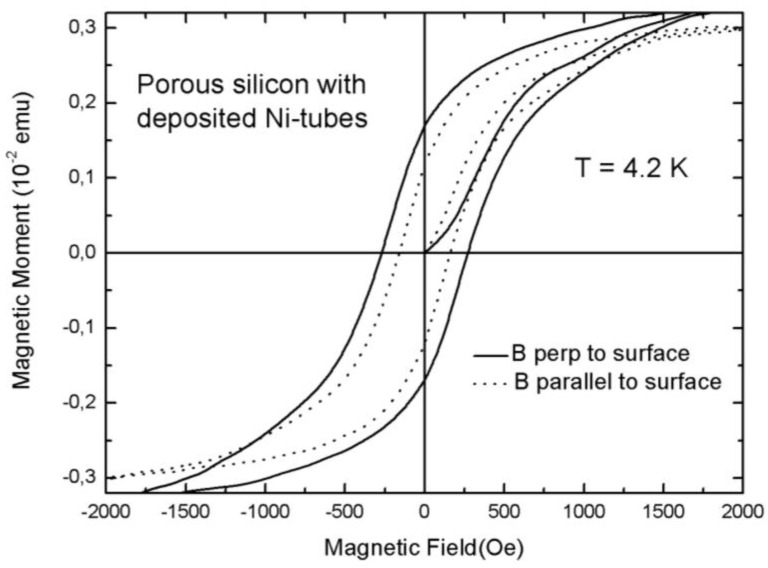
Hysteresis curves of a porous silicon sample with electrodeposited Ni-particles between 2 and 6 nm in size covering the pore walls and forming tube-like arrangements (Figure 14). The measurements have been performed with an applied magnetic field parallel to the pores (full line) and with an applied field perpendicular to the pores (dotted line) [34].

Such a close arrangement of the Ni-particles assures magnetic interactions between them. Due to their size, these small Ni-particles are superparamagnetic but dipolar coupling between them results in a ferromagnetic behavior of the whole system. Magnetic measurements show an anisotropy between easy axis (magnetic field applied along the pores) and hard axis (magnetic field applied perpendicular to the pores) magnetization which corresponds to the behavior of a ferromagnetic metal tube [56]. The measured coercivities are 250 Oe for an applied field parallel to the pores and 170 Oe for a field applied normal to the pores (Figure 15).

Considering the presented semiconducting/ferromagnetic hybrid material, samples with specific magnetic properties can be fabricated whereas the characteristics as coercivity, remanence and anisotropy are tunable in a broad range. Not only ferromagnetic nanostructures with variable size [58], but also small magnetic nanoparticles of a few nanometers in size and even nanotubes consisting of a close arrangement of small particles, can be fabricated. The presented system is of interest due to the combination of semiconducting and ferromagnetic materials, but it is also a good candidate for magnetic sensor or magneto-optical applications especially in the case of integrated devices due to the silicon base material. Furthermore this composite is an interesting candidate for the detection of spin-injection from a ferromagnetic metal into silicon.

## 4. Future Perspectives

Porous silicon with incorporated magnetic nanoparticles is of interest for magnetic/electronic applications especially because the composite system with silicon as substrate is integrable into today’s process technology. The nanocomposite can be fabricated with desired magnetic properties, depending on the geometry and spatial distribution of the deposited nanostructures as well as on the morphology of the porous silicon template. Therefore there is much scope for tailoring the physical properties of the metal/semiconductor nanocompound. This opens a wide field of applications, for example magnetic sensors, magneto-optical devices and utilization in spintronics. Furthermore infiltrated magnetite nanoparticles within the pores of porous silicon offer a nanocomposite system which is a good candidate for biomedical applications. Both materials, Fe_3_O_4_ particles as well as mesoporous silicon are biocompatible and exhibit a low toxicity. Magnetic field-guided drug delivery with nanoscale materials as carriers is a topic of interest, nevertheless there are disadvantages which have to be overcome. On one hand the cytotoxicity of the used materials is under consideration, but on the other, also the behavior of the magnetic particles, for example their intention to agglomerate and the generation of sufficient magnetic force for targeting, is an important issue. A further step of this work is the incorporation of a tandem arrangement of cytocompatible magnetic nanoparticles with potentially therapeutic nucleotides into a readily fabricated porous silicon matrix, which avoids the limitations of existing strategies and promotes sustained delivery of such species that would result in cellular transfection/transduction for sustained periods [59]. Previous efforts from other groups have successfully produced porous Si microparticles with Fe_3_O_4_ surfaces that clearly demonstrated amphiphilic fluidic manipulation and local heating [17,60]. However, nucleotide uptake, delivery, and transfection has not been shown in these materials and the magnetic component is localized along the particle surface, thereby making them susceptible to agglomeration. All in all, the presented nanocomposite, consisting of porous silicon and iron oxide nanoparticles, is a biocompatible system which constrains particle agglomeration and also permits release of therapeutic nucleotide at a targeted cellular environment.

## 5. Conclusions

Metal deposition into the pores of non-magnetic matrices is a widely used method to achieve arrays of nanowires or nanotubes. Porous silicon, a versatile material which is utilizable in various fields, is also a suitable host material for the deposition of ferromagnetic metals. In the presented work, mainly Ni is discussed. Ni can be precipitated in structures with various elongations (arrays of spheres, ellipsoids, wires) whereas the diameters correspond to the pore diameter, as reported by the authors in previous works. Also small superparamagnetic Ni-particles of a few nanometer in size can be deposited on the pore walls of the porous silicon matrix with various packing density. If the particles are precipitated densely packed, *i.e.*, a distance between them in the range of the particle size, a tube-like arrangement of the Ni-particles around the pore walls is achieved. In this case, dipolar coupling between the nanoparticles arises. Magnetization measurements performed with the external field parallel and perpendicular to the tubes display a ferromagnetic behavior and a magnetic anisotropy, which arises due to magnetic interaction between the particles within the pores, whereas the coupling between particles of adjacent pores can be neglected due to the greater distance.

The infiltration of magnetite nanoparticles into the pores of porous silicon also offers interesting magnetic characteristics. The superparamagnetic particles offer an oleic acid coating of a few nanometers which excludes magnetic exchange coupling. If the distance between the particles is close enough, dipolar coupling arises and the blocking temperature T_B_, which indicates the transition between superparamagnetism and a blocked state, can be shifted by the variation of the distance between the particles. Also a magnetic anisotropy is observed between the two magnetization directions, with an applied field parallel and normal to the pores. This behavior is due to the different strength of the coupling between particles inside the pores and between particles of adjacent pores. This magnetite/porous silicon material is an interesting candidate in biomedical research topics because both materials discussed here exhibit low toxicity.
